# Acute Abdomen: A Rare Presentation of Lung Cancer Metastasis

**DOI:** 10.1155/2009/903897

**Published:** 2009-10-15

**Authors:** E. Guérin, O. Gilbert, D. Dequanter

**Affiliations:** ^1^Department of Surgery, CHU Charleroi, Paul Janson Boulevard 92, 6000 Charleroi, Belgium; ^2^Department of Pneumology, CHU Charleroi, Paul Janson Boulevard 92, 6000 Charleroi, Belgium

## Abstract

Surgical emergencies caused by bowel metastases from carcinoma of the lung are very rare. We describe two cases of symptomatic gastrointestinal metastatic small cell carcinoma: the first one concerns a 69-year-old man with an acute abdomen and the second is a 72-year-old man complaining of a gastric ulcer symptoms. We also discuss the current management and the prognosis of these patients.

## 1. Introduction

Abdominal metastases of lung cancer are rare and clinically silent most of the time. Biggest reported series have evaluated gastrointestinal metastases from lung cancer by autopsies: only 12% of patients with lung cancer present gastrointestinal (GI) metastases [1]. According to histological type, the large cell carcinoma accounts for the higher rate of gastrointestinal metastases [2]. Actually, symptomatic intra-abdominal metastases from lung cancer are extremely rare [3]. We describe two cases of symptomatic gastrointestinal metastatic small cell carcinoma: the first one concerns a 69-year-old man with an acute abdomen and the second is a 72-year-old man complaining of a gastric ulcer symptoms.

## 2. Case 1

The first case was a 69-year-old man admitted to the emergency department with an acute abdomen. His past medical history revealed a chronic obstructive and restrictive broncho-pneumopathy. A computed enhanced tomodensitometry (CT) of the abdomen showed the presence of free air and liquid in the peritoneal cavity confirming diagnosis of gastrointestinal (GI) perforation. The patient was planned for an immediate laparotomy. Exploration of the abdominal cavity revealed a purulent peritonitis due to a perforation in the distal jejunum and the presence of a three-centimeter-diameter mass located in the great omentum. The small bowel around the perforation was resected, followed by a jejuno-jejunal side-to-side seromuscular anastomosis to restore the digestive continuity. The omental mass was removed and no other abdominal site was found to be involved peroperatively. A large irrigation with hot saline and adequate drainage ended the procedure. The patient rapidly recovered. Histological analysis of the specimen showed the presence of a parietal necrotic tumor of 1 cm in diameter which was responsible of the perforation. Immunohistochemical analysis revealed the presence of chromogranin, CK7, NSE, synaptophysin, CKAE1/AE3, TTF1 receptors, which was consistent with a small cell carcinoma metastasis. The great omentum's mass had the same histopathological characters. Additional thoracic CT was remarkable for a six-centimeter lung nodule superior to the right hilum with associated multiple mediastinal nodes. A CT-guided biopsy was done and confirmed the neoplastic origin of the primary pulmonary lesion. A close review of the previous abdominal CT showed supracentimetric retroperitoneal nodes and a five-centimeter right adrenal gland metastasis. The patient received chemotherapy (Cisplatin-VP 16). After six cures, the radiological evaluation showed a partial regression of the right hilar mass. The patient received additional chemotherapy.

## 3. Case 2

A 72-year-old man presented with heartburn and epigastric pain at the emergency department. On presentation, physical examination was contributory for an upper quadrant pain on abdominal palpation. Subsequently, biopsies of the ulcer edges were done. After two days, he was admitted to the emergency department with acute epigastric pain. A computed-enhanced tomodensitometry of the abdomen showed the presence of free air in the peritoneal cavity. Exploration of the cavity revealed a perforated gastric ulcer. The histopathological analysis revealed the presence of solid structures with tumoral aspect, containing small tumor cells with a high mitotic index. Immunohistochemical work-up showed the presence of TTF-1, CK 7, synaptophysin, NSE receptors but negative for CK 20, and it was consistent for small cell carcinoma. A thoracic CT showed an anthraco-silicotic pseudotumoral mass in the right lung and a four-centimeter lung nodule superior to the left hilum compatible with a primary lung tumor. An additional abdominal CT was remarkable for left gastric artery infracentimetric nodes and 3 other lesions highly suspected of metastases: one located in the left adrenal gland, one in the splenic hilum, and one in the right bottom in the subcutaneous tissue.

A Positron Emission Tomography (PET) with radiolabeled [18F]-2-fluoro-deoxy-d-glucose (FDG) showed an increased metabolic activity to the head of the pancreas as well as multiple hypermetabolic lesions in the two pulmonary fields and the mediastinum. The patient received chemotherapy (Cisplatine-VP 16) with no response after 6 cures. The patient was eligible for a second line of chemotherapy.

## 4. Discussion

Gastrointestinal (GI) tract manifestation as the first clinical manifestation of a small cell carcinoma is very rare. Furthermore, GI perforation secondary to metastastic lung perforation is extremely rare [4]. Mean age at diagnosis is 64.5 years. There is a male predominance of 89% versus 11% female [3]. Perforation occurred most often in the jejunum (53%) followed by ileum (28%). Combined jejunum-ileum lesions accounted for 4% of perforations. No duodenal perforations were reported [3]. In our case, a jejunal perforation was preoperatively diagnosed. Small perforations were most often caused by adenocarcinoma (23,5%), squamous cell carcinoma (22,7%), large cell carcinoma (20,6%), and a small cell carcinoma, as in our case, in only 19,6% of the cases [3–5]. In some cases, melaena is the first symptom. Bleeding occurred several days before perforation, suggesting that bowel haemorrhage may be a warring of impending rupture [6–8]. However, obstruction is another acute complication described due to metastatic lung carcinoma [9]. Mosier et al. [8] in their study after a review of the literature described only 34 patients. These 34 cases were analysed to clarify the clinical features of the disease. The majority of the patients had a history of abdominal pain (86%), melaena (23%), or nausea and vomiting (26%). Also, 16% of cases had weight loss, 21 patients came to the hospital with perforation and peritonitis, including 9 with lung carcinoma being undiagnosed before laparotomy as in our case. Mostly, lung cancer is previously diagnosed before the diagnosis of small bowel metastasis. In its series, Berger et al. diagnosed lung cancer and treated from 0.5 to 24 months before the diagnosis of small bowel metastasis [10]. Mc Neill performed a study [5] in order to determine the incidence of clinically apparent metastases and also occult metastases of lung cancer to the small intestine; small bowel metastases were present in 46 of the 431 patients with primary lung cancer who underwent autopsy in an eleven-year period. These patients had an average of 4–8 metastatic sites during the same interval, 6 of the 78 patients undergoing small bowel resection for metastatic tumor had lung cancer primaries. No patients survived more than 16 weeks.

Small perforation can occur from metastastic lesion of other primaries. In their study, Ise et al. [11] described two small bowel perforations, one associated with one lymphoma and one with a rhabdomyosarcoma as primary. Indeed, the prognosis is extremely poor. In its series [3, 5, 12], the mean survival was 66 days with in 50% of patients no survival post 30 days. In our study, the patients presented abdominal metastases of lung cancer as first the clinical presentation. The two patients died after, respectively, 10 and 8 months. Survival time was not affected by the therapy to the primary site of the cancer or its metastases [8].

## 5. Conclusion

Acute abdomen as a presentation of lung cancer metastasis is rare and accounts for a poor prognosis. The small bowel is the main location of the GI tract where hemorrhage or perforation occurs. GI metastasis reveals a generalization of the disease as other abdominal sites are probably involved at the time of the laparotomy.
